# Incidence of acute diarrhoea among children (0–59 months old) in Thailand after introduction of rotavirus vaccine: a retrospective national database analysis (2014–2024)

**DOI:** 10.1016/j.lansea.2026.100793

**Published:** 2026-06-03

**Authors:** Thanate Sailuenarm, Wanatpreeya Phongsamart, Wichan Bhunyakitikorn, Pawinee Doungngern, Keswadee Lapphra, Orasri Wittawatmongkol, Supattra Rungmaitree, Nichkamol Lertamornkitti, Nirun Vanprapar, Alan Maleesatharn, Kulkanya Chokephaibulkit

**Affiliations:** a Division of Infectious Diseases, Department of Pediatrics, Faculty of Medicine Siriraj Hospital, Mahidol University, Bangkok, Thailand; b Bureau of Epidemiology, Department of Disease Control, Ministry of Public Health, Nonthaburi, Thailand

**Keywords:** Rotavirus, Vaccine impact, National immunisation program, Acute diarrhoea, Thailand

## Abstract

**Background:**

Rotavirus vaccination has reduced incidence of childhood diarrhoea, but evidence from low- and middle-income countries remains limited. We evaluated diarrhoea incidence and hospitalisation rates among children aged 0–59 months after introduction of rotavirus vaccination in Thailand’s national immunisation programme (February 2020–December 2024).

**Methods:**

We analysed aggregated national surveillance data (ICD-10-TM: A00-A09, K56.1) from Thailand’s Health Data Centre (covering ≈90% of public health facilities), including inpatient, outpatient, and death records. Diarrhoea incidence and hospitalisation rates were assessed using Poisson regression to estimate incidence rate ratios (IRRs) among children 0-59 months of age with acute diarrhoea and among 0–11 months with intussusception. Vaccine impact was assessed by comparing pre-vaccination (2014–2019) and post-vaccination (2021–2024) periods, excluding the year 2020. Analyses were age-stratified as proxies for differential vaccine exposure across birth cohorts under the immunisation program.

**Findings:**

Individual-level demographic characteristics were not available. All-cause and rotavirus-associated diarrhoea incidence declined by 50.0% (IRR 0.50; 95% CI 0.39–0.63) and 52.0% (IRR 0.48; 95%CI 0.39–0.59), respectively, following rotavirus vaccine introduction. Hospitalisation for all-cause and rotavirus-associated diarrhoea decreased by 58.2% (IRR 0.42; 95% CI 0.30–0.58) and 49.0% (IRR 0.51; 95%CI 0.30–0.88), respectively, at 1–4 years post-vaccination. All-cause acute diarrhoea mortality did not differ significantly during pre- and post-vaccination periods (IRR 0.94; 95% CI 0.50–1.77); rotavirus-specific mortalities were too few to analyse. Following vaccine introduction, seasonal peaks between December and February became less pronounced. Intussusception rates remained stable during the post-vaccination period (IRR 1.03; 95% CI 0.82–1.29).

**Interpretation:**

This study demonstrated a significant decline in incidence and hospitalisation of all-cause and rotavirus-associated diarrhoea among children (0–59 months). Sustaining high coverage is essential to maximise public health benefits from vaccination.

**Funding:**

None.

**Research in context**

**Evidence before this study**

We searched PubMed and WHO databases up to December 2024 for studies evaluating rotavirus vaccine impact following national immunisation programme (NIP) introduction in low- and middle-income countries (LMICs). Search terms included “rotavirus vaccine”, “diarrhoea-associated hospitalisation”, “LMIC”, “seasonality”, and “Thailand”. We identified 51 publications, including five systematic reviews, four Thai studies, and 12 impact evaluations from other LMICs. Previous Thai research reported early post-introduction benefits: a pilot study found vaccine effectiveness (VE) of 41% against all-cause diarrhoea hospitalisation and 88% against rotavirus-confirmed diarrhoea, while a 6-year analysis showed 17.8% reduction in outpatient visits, 2.9% in hospitalisations, and 20% in diarrhoea-related mortality among children under five. However, long-term data on incidence, seasonality, herd protection, and intussusception safety beyond the initial rollout remain limited.

**Added value of this study**

Using national surveillance data from 6,571 public health facilities covering inpatient, outpatient, and death records, we compared pre-vaccination (2014–2019) and post-vaccination (2021–2024) periods. All-cause diarrhoea incidence declined by 50.0% and rotavirus-associated diarrhoea by 52.0%. Hospitalisations declined by 58.2% for all-cause and 49.0% for rotavirus-associated diarrhoea. Seasonal peaks became less pronounced. Intussusception rates remained stable. Reductions were also observed in older vaccine-ineligible children.

**Implications of all the available evidence**

Rotavirus vaccine introduction was associated with sustained reductions in childhood diarrhoeal burden in Thailand. Policy implications include maintaining high coverage, considering catch-up vaccination, and integrating rotavirus surveillance with broader pathogen monitoring. Future research in the country should focus on comparing VE by vaccine type, quantifying herd protection thresholds, disentangling pandemic effects, and continued safety monitoring.

## Introduction

Acute diarrhoea remains a leading cause of morbidity and mortality among children, particularly in low- and middle-income countries (LMICs). According to the Global Burden of Disease (2021), diarrheal diseases have resulted in death of over 1 million lives globally across all ages.^1^ Despite progress, diarrhoea still accounts for 9% of mortality among children younger than 5 years, underscoring the urgency for effective prevention.^2^^,^^3^

Globally, rotavirus is the most common cause of severe acute diarrhoea in children and one of the leading causes of high diarrheal mortality rate, especially in Africa, Oceania, and South Asia.^4^ In Thailand, rotavirus was detected in 38.1% of diarrhoea-related hospitalisations,^5^ with the highest burden among infants. Several other studies in the pre-vaccination period have reported a sustained higher proportion of rotavirus positivity among hospitalised patients.^6^^,^^7^

The World Health Organisation (WHO) recommends rotavirus vaccination as the most effective strategy for preventing severe disease.^8^ Given that rotavirus incidence peak between 6 and 10 months of age, the WHO consequently advocates including rotavirus vaccination with diphtheria-tetanus-pertussis (DTP) in national immunisation programmes (NIPs) for infants from 6 weeks of age.^8^^,^^9^ Despite the wide-ranging VE (38.0–98.4%) after its introduction into their NIP,^10^^,^^11^ African, Asian, European, North American, and Oceanian countries consistently reported reduced incidence rates of rotavirus-associated diarrhea,^12^^,^^13^ hospitalisation,^12^^,^^14^, ^15^, ^16^, ^17^, ^18^ and mortality.^15^^,^^19^ Additionally, vaccine introduction was also found to influence the seasonal dynamics, delaying onset and attenuating the intensity of annual epidemic peaks.^14^^,^^20^ It is unclear whether natural rotavirus infection increases the risk of intussusception as a complication^21^ or if there is no significant association.^22^, ^23^, ^24^ Therefore, surveillance for intussusception rates is also considered an important outcome to monitor alongside VE.

In February 2020, Thailand introduced monovalent rotavirus vaccines (Rotarix, GlaxoSmithKline Biologicals, London, United Kingdom) on a 2-dose schedule (2, 4 months) into the NIP following a pilot study in 2012 that demonstrated VE in children between 2 and 18 months of age, with reductions of 41% in hospitalisations due to all-cause acute diarrhoea and 88% for rotavirus-associated diarrhoea, along with evidence of herd protection.^12^ Charoenwat et al.^25^ performed a 6-year analysis (2015–2020) of the population-level impact following its implementation and found early benefits of a 17.8% reduction in outpatient visits, 2.9% in hospitalisation, and 20% in diarrhoea-related mortality among children younger than five years.^25^ Since its introduction, the program transitioned to distributing pentavalent vaccines (RotaTeq, Merck Sharp & Dohme Corp, New York, United States) on a 3-dose schedule (2, 4, and 6 months) in August 2021, and reverted to distributing monovalent vaccines on February 2023 onwards. Rotavirus vaccination eligibility covered infants under 15 weeks of age at the time of their first dose and 32 weeks of age at the time of their final dose; no catch-up vaccinations were implemented. The first dose was recommended at 2 months and was generally administered with the first dose of a DTP-containing vaccination.

This study assesses the long-term, population-level impact of rotavirus vaccine introduction in Thailand’s NIP by utilising national surveillance data. Pre- (2014–2019) and post-vaccination (2021–2024) periods are compared to evaluate changes in the incidence of all-cause acute diarrhoea, rotavirus-associated acute diarrhoea, hospitalisations and mortality, seasonal trends, herd effects in unvaccinated age groups, and intussusception rate. Our findings aim to inform national immunisation policies, guide future vaccine strategies, and provide knowledge on the impact of rotavirus vaccines in a middle-income country setting.

## Methods

This retrospective, population-based ecological study utilised national health statistics data from January 1, 2014 to December 31, 2024, obtained from the Medical and Health Data Centre (HDC), Ministry of Public Health (MoPH), Thailand. Diagnoses were identified using Thai-modified International Statistical Classification of Diseases and Related Health Problems, Tenth Revision (ICD-10-TM) codes A00-A09 for acute diarrhoea and K56.1 for intussusception. These data were recorded across both outpatient departments (OPD) and inpatient departments (IPD) using standardised diagnostic coding^26^ implemented across public health facilities, supported by the methodology of Charoenwat et al.^27^ and Fernandes et al.^28^ Data were derived from public healthcare facilities at all levels, including subdistrict health-promoting, community, general, and regional hospitals, representing 6,571 of 7,344 (89.96%) health facilities across 13 health-service regions. The database does not include university hospitals or private healthcare facilities; however, the public health system is the primary provider of paediatric care, particularly for common conditions such as acute diarrhoea. Age-specific population denominators were obtained from the same database to calculate incidence rates of diarrhoea, hospitalisations, and deaths.

This study included children between 0-59 months of age with acute diarrhoea and 0–11 months with intussusception recorded in the HDC database. All-cause acute diarrhoea (A00-A09) encompassed specific bacterial pathogens–cholera (A00), typhoid and paratyphoid fever (A01), other Salmonella infections (A02), shigellosis (A03), other bacterial intestinal infections (A04), other bacterial foodborne intoxications (A05), protozoal pathogens–amoebiasis (A06), other protozoal intestinal diseases (A07), viral and other specified intestinal infection (A08), and diarrhoea and gastroenteritis of presumed infectious origin (A09). Rotavirus-associated acute diarrhoea was defined by an ICD-10-TM code of A08.0, and intussusception cases by K56.1. The use of A08 and A09 as proxies for acute diarrhoea in young children is supported by Charoenwat et al.,^27^ who analysed diarrheal diagnoses among Thai children younger than 5 years using national health data from 2015 to 2019 and demonstrated the reliability of these codes as indicators of acute diarrheal disease burden, particularly for evaluating the population-level impact of rotavirus vaccination. While the HDC database provides aggregated case without linked laboratory results, pathogen-specific ICD-10-TM codes are generally assigned when laboratory evidence is available, supporting the use of rotavirus-specific diagnoses in this study.

For vaccine impact analysis, the pre-vaccination period was defined as January 1, 2014 to December 31, 2019 and the post-vaccination period was defined as January 1, 2021 to December 31, 2024. The year 2020 was excluded as a transition period during vaccine implementation. Based on previous national data, the average pre-vaccination hospitalisation rate for diarrhoea was 31.4 per 1,000 children under five years.^25^ Assuming a 41% rate reduction post-vaccination,^12^^,^^27^ a two-sided test, 95% confidence, and 80% power yielded a minimum required sample size of 10,112 cases (5,056 per group). Given the use of nationwide data, all available cases were included in the analysis which ensured sufficient power to detect meaningful differences.

### Statistical analyses

Annual case counts were stratified by age group and ICD-10-TM diagnosis codes from OPD, IPD, and death records, and analysed alongside age-specific population denominators from the HDC. Annual incidence rates of all-cause and rotavirus-associated diarrhoea were calculated as the number of cases divided by the corresponding annual population and expressed per 1,000 children 0–59 months of age. Intussusception incidence was calculated per 1,000 children 0-11 months of age, and mortality rate from all-cause acute diarrhoea was expressed per 1,000,000 children age 0–59 months. The mortality rate of rotavirus-associated acute diarrhoea could not be separately analysed due to the small number of cases. OPD and IPD records were combined to estimate overall incidence, while IPD data alone were used for hospitalisation rates. Mortality analyses were based on death records. Annual incidence values were summarised using the medians and interquartile ranges (IQRs) for the pre- and post-vaccination periods. Incidence rate ratios (IRRs) and 95% confidence intervals (CIs) were used to compare disease burden between periods. IRRs were calculated by dividing the pooled post-vaccination incidence rate by the pooled pre-vaccination incidence rate, based on the total cases and total population in each period. Two-sided *p*-values were computed, and vaccine impact was calculated as (1-IRR)×100. Subgroup analyses were performed for children 0-11 months of age, representing a high-risk population, to evaluate both overall incidence and hospitalisation outcomes.

Annual national rotavirus vaccine and DTP1 coverage estimates among children 0–59 months of age were obtained from routine immunisation coverage reports from the HDC, MoPH. DTP1 coverage is shown as comparison as both vaccines are administered during infancy through the NIP schedule. Coverage was defined as the proportion of children who received full vaccine series per Thailand’s NIP.

Poisson regression models were fitted using annual case counts as the outcome and the annual population as an offset (log scale); robust standard errors were applied in all models. For all-cause acute diarrhoea outcomes, the models were additionally adjusted for calendar year (included as a continuous variable) to account for underlying secular trends. For rotavirus-specific, intussusception, and mortality outcomes, model without calendar year adjustment were used as the primary analyses due to the relatively small number of events, resulting in imprecise estimates. To evaluate both direct and indirect (herd) effects, analyses were stratified by age groups based on vaccine eligibility under the NIP, which expanded over time following vaccine introduction. Younger age groups corresponding to vaccine-eligible cohorts in each year were compared across periods to assess the direct effects, while older age groups unlikely to have received the vaccine were used as comparison groups for herd effects. Year-specific cohorts were analysed (e.g., children aged 0–23 and 24–59 months in 2021, 0–35 and 36–59 months in 2022, and 0–47 and 48–59 months in 2023) and compared with corresponding age groups in the pre-vaccination period. Monthly incidence rates were calculated using monthly case counts divided by the corresponding annual population and were used to assess seasonal trends. Analyses were conducted using Python (version 3.13.6). Pandas (version 2.3.1) was used for data preprocessing and statsmodels (version 0.14.5) was used for statistical analysis. Statistical significance was defined as *p* < 0.05. Seasonal trends were visualised using Excel line charts to illustrate long-term disease burden patterns.

### Ethics statement

Formal permission to access and analyse national-level health data was submitted and approved by the HDC. Ethical approval was obtained from the Siriraj Institutional Review Board, Faculty of Medicine Siriraj Hospital, Mahidol University (COA no. Si 642/2567, 13 September 2024). Informed consent was waived due to the retrospective and anonymised nature of the data.

### Role of the funding source

No funding was secured for this study. Therefore, no funders had any role in study design, data collection, data analysis, interpretation, or writing of the report.

## Results

The analysis included aggregated annual case counts of acute diarrhoea from the HDC database, representing data from 6,571 public health facilities. Individual-level demographic characteristics were not available in this dataset. National rotavirus vaccine coverage increased progressively since its introduction in February 2020, reaching 59.3% in 2021 and 76.5% in 2024, whereas DTP1 coverage remained consistently above 90% throughout the study period, reflecting sustained NIP performance (Supplementary Fig. S1).

The median annual incidence rate for all-cause acute diarrhoea among children (0–59 months) decreased from 357.06 to 216.23 per 1,000 children, with a vaccine impact of 50.0.% (IRR 0.50 [95% CI 0.39–0.63]; *p* < 0.0001). The vaccine impact was 52.0% (IRR 0.48 [95% CI 0.39–0.59]; *p* < 0.0001) for rotavirus-associated acute diarrhoea (Table 1, Supplementary Figs. S2 and S3).

**Table 1:** Incidence rates of all-cause acute diarrhoea, rotavirus-associated diarrhoea, hospitalisations, and mortality in children aged 0–59 months across pre- and post-vaccination periods.

Outcome	Pre-vaccination period (2014–2019), Median (IQR) IR per 1000 children 0–59 months	Post-vaccination period (2021–2024), Median (IQR) IR per 1000 children 0–59 months	IRR (95% CI)	Vaccine impact^b^ (95% CI)	*p* value
All-cause acute diarrhoea^a^	357.06 (342.23–382.20)	216.23 (199.12–221.71)	0.50 (0.39–0.63)	50.0% (36.9–60.4)	<0.0001
Rotavirus-associated diarrhoea^a^	1.43 (1.31–1.62)	0.73 (0.66–0.79)	0.48 (0.39–0.59)	52.0% (41.5–60.7)	<0.0001
Hospitalisation due to all-cause acute diarrhoea	202.44 (191.77–220.30)	80.29 (67.32–98.79)	0.42 (0.30–0.58)	58.2% (42.5–69.7)	<0.0001
Hospitalisation due to rotavirus-associated diarrhoea	0.12 (0.07–0.18)	0.07 (0.06–0.08)	0.51 (0.30–0.88)	49.0% (12.3–70.4)	0.015
Mortality from all-cause acute diarrhoea	8.76 (4.93–13.20)	9.64 (7.87–10.83)	0.94 (0.50–1.77)	5.6% (−77.2–49.7)	0.857

Most diarrhoea cases in Thailand were not routinely tested for rotavirus, and were mostly coded as non-laboratory-confirmed ICD-10-TM. This potentially underestimates the true burden of rotavirus-associated diarrhoea.

Abbreviations: IR, Incidence Rate; IRR, Incidence Rate Ratio.

a Incidence rates include both outpatient and inpatient cases.

b Vaccine impact was calculated from (1−IRR) × 100%.

One-to-four years post-vaccination, median annual incidence rate of hospitalisation decreased from 202.44 to 80.29 per 1,000 children, with a vaccine impact of 58.2% (IRR 0.42 [95% CI 0.30–0.58]; *p* < 0.0001) for all-cause acute diarrhoea. The vaccine impact was 49.0% (IRR 0.51 [95% CI 0.30–0.88]; *p* = 0.015) for rotavirus-associated hospitalisation (Table 1, Supplementary Figs. S4 and S5). No significant difference was observed in median annual mortality rate between two periods (IRR 0.94 [95% CI 0.50–1.77]; *p* = 0.857), (Table 1, Supplementary Fig. S6).

In subgroup analyses of children belonging to 0–11 months, the incidence and hospitalisation of all-cause acute diarrhoea decreased significantly with a vaccine impact of 45.7% (IRR 0.54 [95% CI 0.48–0.61]; *p* < 0.0001) and 53.6% (IRR 0.46 [95% CI 0.33–0.66]; *p* < 0.0001), respectively (Table 2, Supplementary Figs. S7 and S8).

**Table 2:** Incidence rates of all-cause acute diarrhoea and hospitalisations in children aged 0–11 months across pre- and post-vaccination periods.

Outcome	Pre-vaccination period (2014–2019), Median (IQR) IR per 1000 children 0–11 months	Post-vaccination period (2021–2024), Median (IQR) IR per 1000 children 0–11 months	IRR (95% CI)	Vaccine impact^b^ (95% CI)	*p* value
All-cause acute diarrhoea^a^	525.81 (498.73–557.04)	328.51 (308.83–344.44)	0.54 (0.48–0.61)	45.7% (39.1–51.7)	<0.0001
Hospitalisation due to all-cause acute diarrhoea	304.46 (282.48–326.64)	135.89 (101.89–178.32)	0.46 (0.33–0.66)	53.6% (34.5–67.1)	<0.0001

Abbreviations: IR, Incidence rate; IRR, Incidence rate ratio.

a Incidence rates include both outpatient and inpatient cases.

b Vaccine impact was calculated from (1−IRR) × 100%.

Analyses across different years in post vaccination period for the years 2021, 2022, and 2023 for various age cohorts, showed significant reductions in all-cause acute diarrhoea incidence and hospitalisation in both younger and older age groups (Table 3, Supplementary Figs. S9 and S10).

**Table 3:** Incidence rates of all-cause acute diarrhoea and hospitalisations in children in the age group covered by vaccination and not covered by vaccination program.

Year	Age group^a^ (months)	Outcome	Pre-vaccination period (2014–2019) Median (IQR) IR per 1000 children	Post-vaccination period IR per 1000 children^c^	IRR (95% CI)	Vaccine impact^d^ (95% CI)	*p* value
2021	0–23	All-cause acute diarrhoea^b^	549.82 (527.26–572.06)	266.57	0.47 (0.43–0.50)	53.5% (49.7–57.0)	<0.0001
	24–59	All-cause acute diarrhoea^b^	238.35 (226.12–267.91)	94.74	0.35 (0.31–0.41)	64.8% (59.5–69.4)	<0.0001
	0–23	Hospitalisation due to all-cause acute diarrhoea	313.83 (297.39–332.54)	153.82	0.45 (0.42–0.49)	54.8% (51.1–58.2)	<0.0001
	24–59	Hospitalisation due to all-cause acute diarrhoea	133.90 (125.50–151.74)	53.64	0.34 (0.30–0.40)	65.6% (60.3–70.3)	<0.0001
2022	0–35	All-cause acute diarrhoea^b^	469.87 (454.97–497.26)	280.54	0.56 (0.51–0.62)	44.0% (38.3–49.2)	<0.0001
	36–59	All-cause acute diarrhoea^b^	195.48 (181.58–225.98)	141.36	0.59 (0.49–0.72)	40.8% (28.4–51.0)	<0.0001
	0–35	Hospitalisation due to all-cause acute diarrhoea	267.75 (255.44–287.88)	161.56	0.54 (0.49–0.60)	45.8% (40.1–51.0)	<0.0001
	36–59	Hospitalisation due to all-cause acute diarrhoea	109.56 (101.58–127.15)	80.05	0.58 (0.47–0.70)	42.4% (29.8–52.7)	<0.0001
2023	0–47	All-cause acute diarrhoea^b^	406.31 (390.75–434.89)	253.29	0.57 (0.50–0.64)	43.0% (35.6–49.6)	<0.0001
	48–59	All-cause acute diarrhoea^b^	166.98 (156.77–191.88)	144.95	0.67 (0.55–0.87)	31.1% (13.3–45.2)	0.001
	0–47	Hospitalisation due to all-cause acute diarrhoea	230.76 (219.29–251.19)	78.80	0.30 (0.26–0.34)	70.4% (66.4–73.9)	<0.0001
	48–59	Hospitalisation due to all-cause acute diarrhoea	93.28 (87.40–107.62)	42.72	0.35 (0.28–0.44)	65.2% (55.9–72.5)	<0.0001

Abbreviations: IR, Incidence Rate; IRR, Incidence Rate Ratio.

a Age groups were defined by year to approximate differences in vaccine exposure across birth cohorts following rotavirus vaccine introduction in 2020 without catch-up vaccination.

b Incidence rates include both outpatient and inpatient cases.

c Post-vaccination values represent single-year incidence rates across post-vaccination periods.

d Vaccine impact was calculated from (1−IRR) × 100%.

**Fig. 1:**
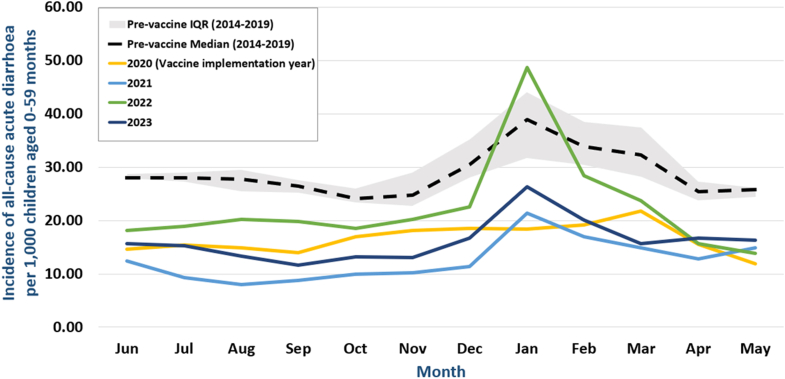
**Seasonality in incidence of all-cause acute diarrhoea during pre-vaccination (2014–2019) and post-vaccination (2021–2024) periods.** Monthly incidence was aligned using a June–May seasonal year (e.g., 2020 represents June 2020–May 2021). The shaded area represents the IQR, and the dashed line represents the median during the pre-vaccination period. Abbreviation: IQR, Interquartile Range.

**Fig. 2:**
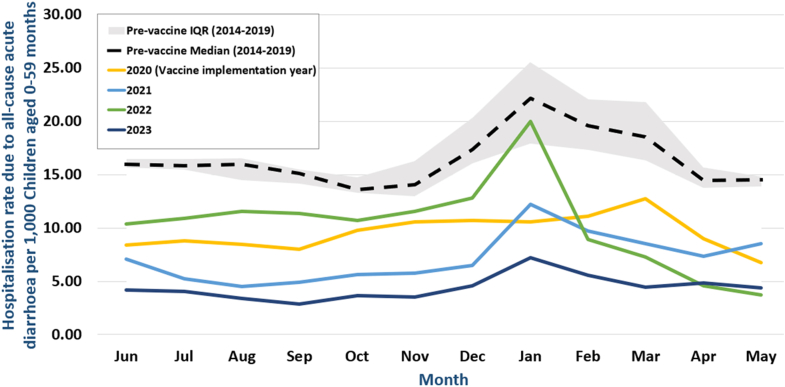
**Seasonality of hospitalisation due to all-cause acute diarrhoea during pre-vaccination (2014–2019) and post-vaccination (2021–2024) periods.** Monthly hospitalisations were aligned using a June–May seasonal year (e.g., 2020 represents June 2020 to May 2021). The shaded area represents the IQR, and the dashed line represents the median during the pre-vaccination period. Abbreviation: IQR, Interquartile range.

During the pre-vaccination period, the incidence of all-cause acute diarrhoea exhibited consistent seasonal peaks from December to February, with the highest burden observed in January. Following vaccine introduction, this trend was less pronounced, and no consistent peak observed during the post-vaccination period. A similar pattern was observed for hospitalisation due to all-cause acute diarrhoea, with diminished seasonal peaks in the post-vaccination period (Fig. 1, Fig. 2).

The incidence of intussusception in children 0-11 months did not differ between the pre- and post-vaccination periods, with a median of 0.98 per 1,000 children (IQR: 0.84–1.09) during the pre-vaccination period and 0.94 (IQR: 0.90–1.02) per 1,000 children in the post-vaccination period (IRR 1.03 [95% CI 0.82–1.29]; *p* = 0.788) (Table 4, Supplementary Fig. S11).

**Table 4:** Incidence rate of intussusception in children aged 0–11 months across pre- and post-vaccination periods.

Outcome	Pre-vaccination period (2014–2019), Median (IQR) IR per 1000 children 0–11 months	Post-vaccination period (2021–2024), Median (IQR) IR per 1000 children 0–11 months	IRR (95% CI)	Vaccine impact^a^ (95% CI)	*p* value
Intussusception	0.98 (0.84–1.09)	0.94 (0.90–1.02)	1.03 (0.82–1.29)	−3.1% (−28.9–17.5)	0.788

Abbreviations: IR, Incidence rate; IRR, Incidence rate ratio.

a Vaccine impact was calculated from (1−IRR) × 100%.

## Discussion

This study provided country-specific evidence on incidence, hospitalisation, and mortality due to all-cause diarrhoea and rotavirus-associated diarrhoea among 0-59-month-old children in Thailand using national surveillance data. Trends during pre- and post-rotavirus vaccine introduction periods were compared and showed a significant decline along with loss of seasonality. Intussusception rates remained stable 1–4 years after rotavirus vaccination introduction. These findings reflect direct, and potential indirect, impacts from rotavirus vaccination. However, community based longitudinal studies, along with robust surveillance mechanism, are needed to understand the complete impact of vaccination.

With Thailand’s estimated vaccine coverage of 84% (72%–91%),^29^ the impact observed were similar to those reported from countries like South Africa and the United States.^30^, ^31^, ^32^ Rotavirus vaccine coverage during the study period remained lower than DTP1 coverage (which consistently exceeded 90%), primarily due to age restrictions that limited eligibility to infants younger than 15 weeks for the first dose and 32 weeks for the final dose. Infants presenting outside these narrow windows were ineligible for rotavirus vaccination through the NIP. In 2025, following the relaxation of these age restrictions, rotavirus vaccine coverage increased to 82.6%, approaching DTP coverage levels and potentially enhancing future population-level impact. The magnitude of reduction observed in this study is consistent with previous reports from multiple regions, though our findings were lower than Australia’s reduction of 46% and 85% for non-rotavirus- and rotavirus-associated hospitalisation.^16^ These discrepancies may stem from differences in vaccine coverage, healthcare access, treatment practice, or diagnosis practices.

Tharmaphornpilas et al.^12^ (2017) previously conducted a Thai pilot VE study across two provinces, Sukhothai and Phetchabun, and observed a 41% and 88% VE in all-cause acute diarrhoea hospitalisations reduction and rotavirus-confirmed diarrhoea prevention for vaccinated children.^12^ The modest difference in effect size between the previously reported VE and the observed vaccine impact is methodologically appropriate and epidemiologically informative. VE reflects the direct protection in vaccinated individuals under controlled settings, whereas vaccine impact at the population-level incorporates both direct and indirect effects, representing real-world implementation that includes coverage variations, vaccination schedule adherences, and the presence of unvaccinated individuals.

However, in this study substantial reductions were also observed in children 0-11 months of age, a group at highest risk for severe diarrheal disease. In this subgroup, both incidence and hospitalisation due to all-cause acute diarrhoea declined markedly following vaccine introduction, highlighting the importance of vaccination in protecting the vulnerable population.

Previous studies reported reduced infections and hospitalisations among older children ineligible for rotavirus vaccination, likely due to long-term herd protection conferred by younger vaccinated populations.^12^^,^^15^^,^^16^ Similarly, this study observed suggested protection in older children through reduced incidence and hospitalisation rates due to all-cause acute diarrhoea; comparable to those demonstrated in Australia and the United States adults.^16^^,^^33^ These effects became more evident over time, with progressively greater reductions observed as the proportion of vaccine-eligible cohorts expanded following its implementation. This finding underscores the broader community benefit of vaccination beyond direct protection conferred to vaccinated individuals. However, these reductions may not be attributed solely to vaccination. Despite the sustained decline in infections and hospitalisations following vaccine introduction, this decline coincided with the COVID-19 pandemic where contemporaneous (e.g., behavioural changes or altered transmission dynamics) and confounding factors (e.g., inherent disease cycles or acquired natural immunity from prior infections) may have influenced disease trends and hospital visits. As accurately estimating indirect protection requires complex assumptions about disease transmission and population immunity, future studies should standardise metrics, age categorisations, and analytical timeframes to improve comparability. Nonetheless, early and widespread vaccine coverage among children remains crucial to reducing infection burden across all age groups.

Prior to the vaccine’s implementation in the NIP, infections in Thailand^12^ and several other countries^34^ typically peaked during winter (i.e., December–March). Post-rollout, diminished diarrheal illness seasonality and wintertime peak severity were reported.^16^^,^^31^^,^^35^ This pattern, with shifted and diminished seasonality for acute diarrhoea incidence during the post-vaccination period among children younger than five, was observed in our study as well. These changes may also heavily reflect the COVID-19-related mobility restrictions and hygiene practices, rather than vaccination effectiveness and herd protection alone. However, a transient increase in incidence was observed in 2023, with a resurgence of seasonal peaks. This pattern may be explained by concurrent outbreaks of other enteric pathogens, particularly norovirus, which was reported by communicable disease surveillance system as the predominant cause of acute diarrheal illness in Thailand during this period.^36^ Despite this increase in incidence, hospitalisation rates remained substantially lower than in the pre-vaccination period, suggesting sustained protection against severe disease.

During the COVID-19 pandemic, decreased incidence and hospitalisation rates for rotavirus-associated diarrhoea and other intestinal pathogens were observed across many countries.^34^^,^^37^, ^38^, ^39^, ^40^, ^41^ Concurrently, shortened^38^ and shifted^42^ seasonal patterns of rotavirus infections were also reported. This effect was likely attributable to the adoption of nonspecific protective measures (e.g., lockdowns, physical distancing, handwashing, and mask usage), which inadvertently altered the transmission and seasonal dynamics of viruses.^39^^,^^42^^,^^43^ Subsequently, studies noted increased rotavirus incidence^44^^,^^45^ and emergency department visits^42^ after the pandemic. This possibly reflected an immunity debt among rotavirus-naïve children following reduced exposure as social distancing practices relaxed and vaccine coverage decreased during 2022–2023. Despite this slight incidence increase, the rates remained well below pre-pandemic levels,^27^ underscoring the sustained population-level impact of rotavirus vaccination.

Although rotavirus vaccinations carry a small excess risk of intussusception,^46^^,^^47^ the WHO deems this risk minor to its overall benefit.^47^ This study did not observe an increase in intussusception incidence following vaccine introduction. This finding is consistent with the favourable risk–benefit profile reported globally. However, changes in healthcare utilisation and disease transmission patterns during the COVID-19 pandemic may also have influenced these observations.^48^, ^49^, ^50^

This study had some limitations. The HDC database did not include private or university hospitals, potentially underestimating disease burden. Regardless, vaccine impact may be determined using the database for pre- and post-vaccine rollout comparison. The analysis was based on aggregated data and did not include individual-level demographic characteristics (e.g., sex, ethnicity, or clinical severity), individual vaccination histories, and regional or temporal variations in vaccine coverage. This limited our ability to perform detailed subgroup analyses, including assessments and comparisons of monovalent and pentavalent rotavirus vaccine impacts. The analyses were limited to principal diagnoses, potentially missing diarrhoea cases recorded as secondary conditions or nosocomial-acquired rotavirus-associated diarrhoea. Most diarrhoea cases in Thailand were not tested for rotavirus, resulting in diagnoses based on non-laboratory-confirmed ICD-10-TM codes. Relying solely on the laboratory-confirmed rotavirus potentially underestimates the true burden due to fluctuations in testing availability and clinician behaviours over time. To account for this, our analysis assessed all-cause acute diarrhoea in parallel with rotavirus associated diarrhoea. Nevertheless, the use of standardised coding across pre- and post-vaccination periods ensured consistent case definitions and international research comparability, supporting national public health policy evaluation. Additionally, as diagnostic and coding practices did not change systematically following vaccine introduction, even though the absolute incidence is underestimated, the observed vaccine impact is unlikely to be substantially biased. The COVID-19 pandemic overlapped with the study period. As such, pandemic-related public health interventions may have independently influenced the rotavirus vaccine impact, especially during stringent measures in 2021. Despite the overlap, robust temporal trend analyses were possible due to the extended pre- and post-vaccination comparison periods. Furthermore, the sustained reduction in disease incidence following the lifting of major restrictions in 2022 suggests that this effect was not purely driven by pandemic-related behavioural changes.

In conclusion, this study on national database analyses demonstrates significant decline in incidence, hospitalisation, and mortality rates of all-cause and rotavirus-associated diarrhoea in children (0–59 months) following introduction of rotavirus vaccination in Thailand, with herd protection potentially extending to older unvaccinated children. Diminished seasonality and wintertime peak in incidence was observed with no change in incidence of intussusception. These findings indicate potential indirect impact from the scale up of rotavirus vaccination. However, continued long term surveillance is required to assess population level impacts. It is essential to continue monitoring mortality trends, adverse events, and evolving disease burden, while also conducting future research to assess VE by vaccine type and evaluate age-specific herd protection. This would inform national policy decisions, such as optimizing vaccine schedules, implementing catch-up campaigns for under-vaccinated cohorts, and integrating rotavirus surveillance with broader enteric pathogen monitors–further strengthening immunisation planning and long-term public health impact in Thailand.

## Contributors

TS, WP, and KC conceptualised the study and designed the methodology. WB and PD acquired access to the national surveillance data. TS drafted and edited the published work. KL, OW, SR, NL, and NV contributed to the investigation and edited the published work. TS and AM curated, managed, and analysed the data. WP and KC facilitated and provided administrative support for the study. TS and WP have accessed and verified all data reported in the study. All authors read and approved the final manuscript.

## Data sharing statement

The data used in this study were obtained from the Health Data Centre, Ministry of Public Health, Thailand and made available by the Division of Digital Disease Control and Bureau of Epidemiology, Department of Disease Control, Ministry of Public Health, Thailand. Public access is restricted, but may be granted upon reasonable request.

## Declaration of interests

The authors have no conflicts of interest to disclose.

## Appendix A Supplementary data

Supplementary Figs. S1–S11

Translated Abstract
