# Supplementary material for: Incidence of acute diarrhoea among children (0–59 months old) in Thailand after introduction of rotavirus vaccine: a retrospective national database analysis (2014–2024)
Source: Lancet Reg Health Southeast Asia. 2026 Jun 3;50:100793. doi: 10.1016/j.lansea.2026.100793 (PMC13254665; doi:10.1016/j.lansea.2026.100793)
Supplement: Supplementary Figs. S1–S11 [file mmc1.docx]

**Supplementary Results**

**Incidence of acute diarrhoea among children (0-59 months old) in Thailand after national introduction of Rotavirus vaccine: a retrospective national database analysis (2014-2024)**

**Table of contents**

**Supplementary Figure S1.** Annual national coverage of rotavirus vaccines and DTP1 in Thailand between 2020 to 2024.

**Supplementary Figure S2.** Incidence of all-cause acute diarrhoea in children aged 0-59 months.

**Supplementary Figure S3.** Incidence of rotavirus-associated diarrhoea in children aged 0-59 months.

**Supplementary Figure S4.** Incidence of hospitalisation due to all-cause acute diarrhoea in children aged 0-59 months.

**Supplementary Figure S5.** Incidence of hospitalisation due to rotavirus-associated diarrhoea in children aged 0-59 months.

**Supplementary Figure S6.** Mortality from all-cause acute diarrhoea in children aged 0-59 months.

**Supplementary Figure S7.** Incidence of all-cause acute diarrhoea in children aged 0-11 months.

**Supplementary Figure S8.** Incidence of hospitalisation due to all-cause acute diarrhoea in children aged 0-11 months.

**Supplementary Figure S9.** Incidence of all-cause acute diarrhoea in children by age group covered by vaccination and not covered by vaccination program.

**Supplementary Figure S10.** Incidence of hospitalisation due to all-cause acute diarrhoea by age group covered by vaccination and not covered by vaccination program.

**Supplementary Figure S11.** Incidence of intussusception in children aged 0-11 months

**Supplementary Figure S1.** Annual national coverage of rotavirus vaccines and DTP1 in Thailand between 2020 to 2024.


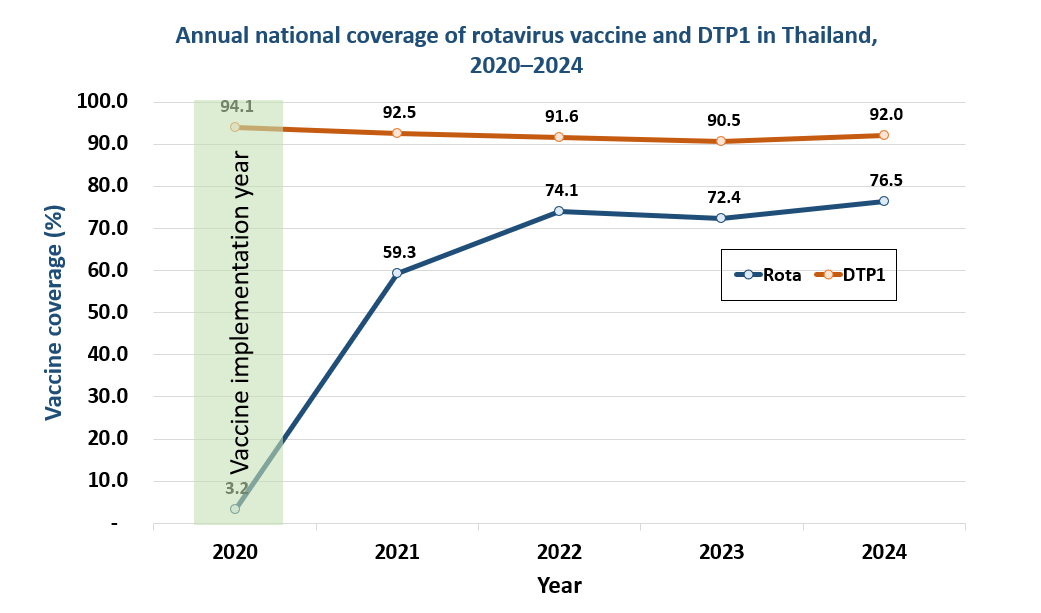


**Supplementary Figure S2.** Incidence of all-cause acute diarrhoea in children aged 0-59 months.

**
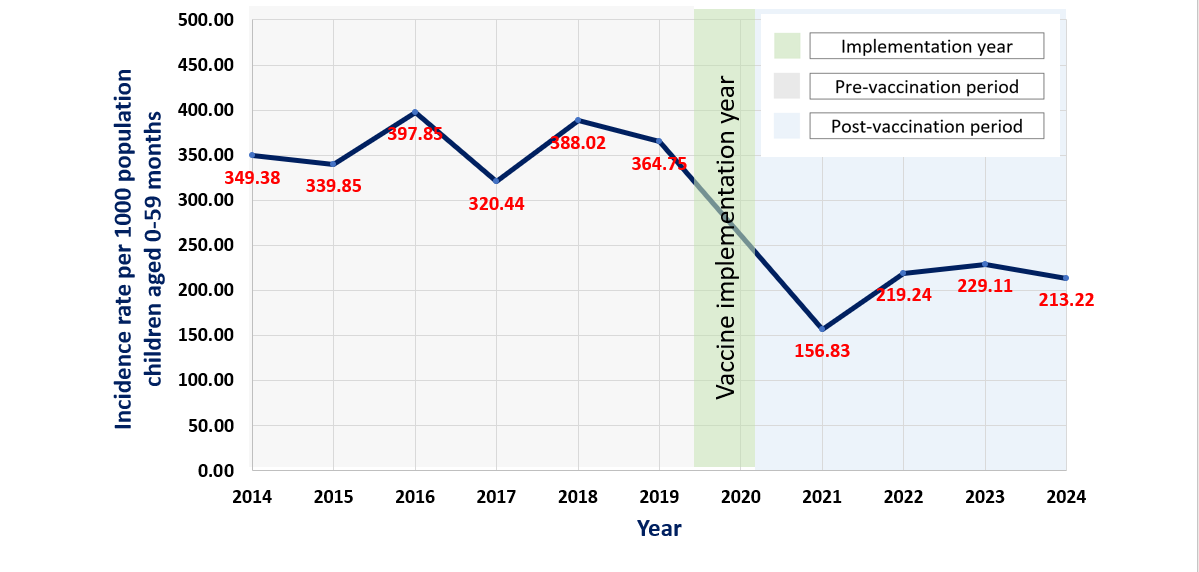
**

**Supplementary Figure S3.** Incidence of rotavirus-associated diarrhoea in children aged 0-59 months.

**
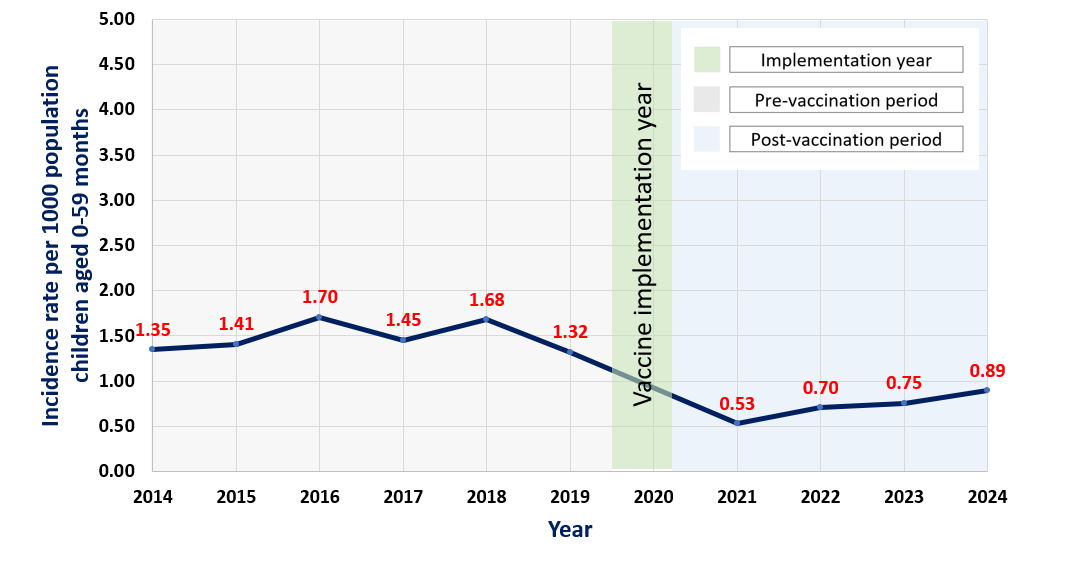
**

**Supplementary Figure S4.** Incidence of hospitalisation due to all-cause acute diarrhoea in children aged 0-59 months.


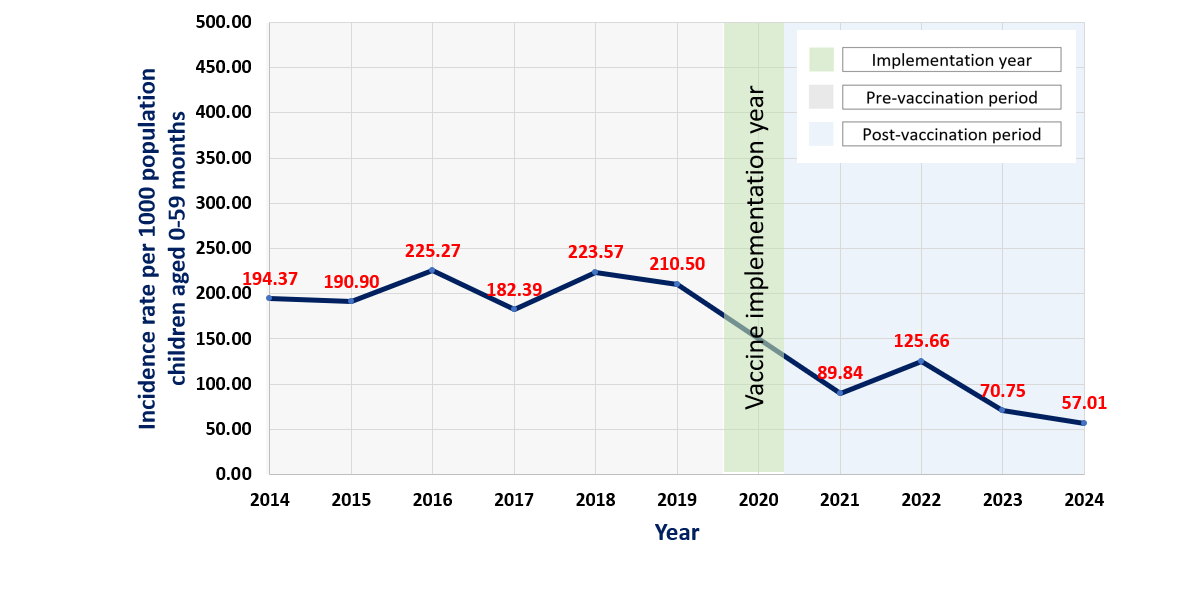


**Supplementary Figure S5.** Incidence of hospitalisation due to rotavirus-associated diarrhoea in children aged 0-59 months.


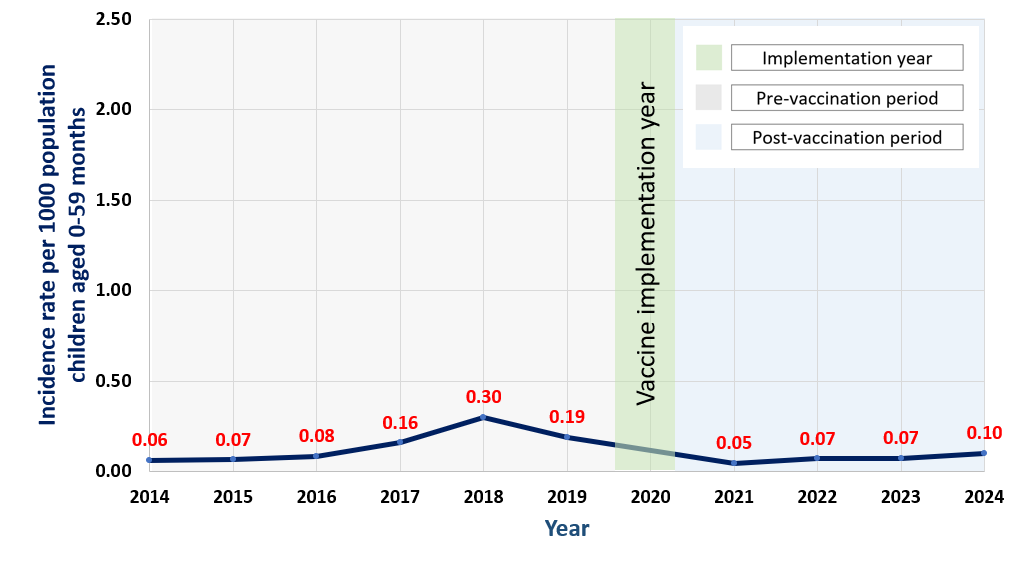


**Supplementary Figure S6.** Mortality from all-cause acute diarrhoea in children aged 0-59 months.

**
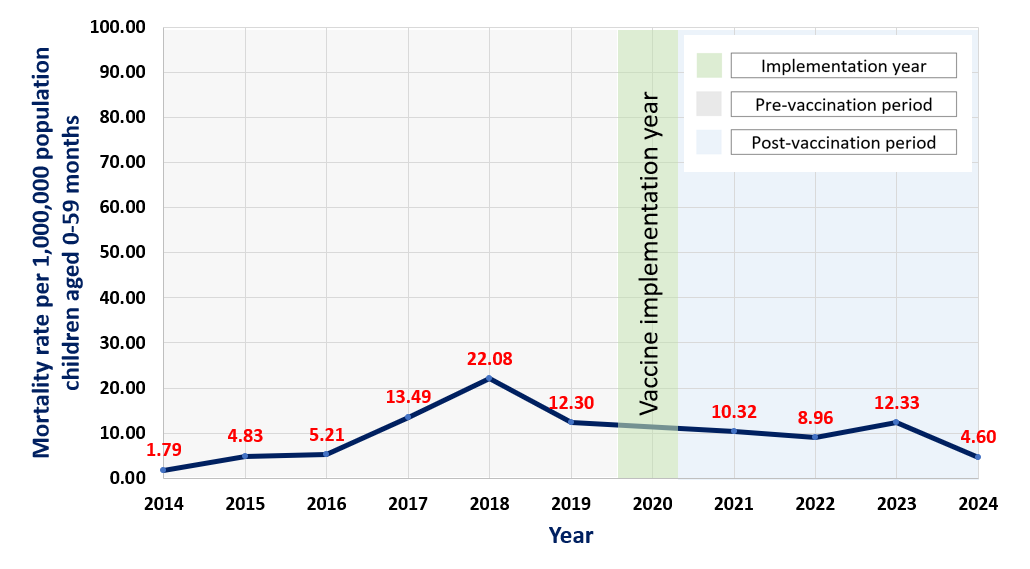
**

**Supplementary Figure S7.** Incidence of all-cause acute diarrhoea in children aged 0-11 months.

**
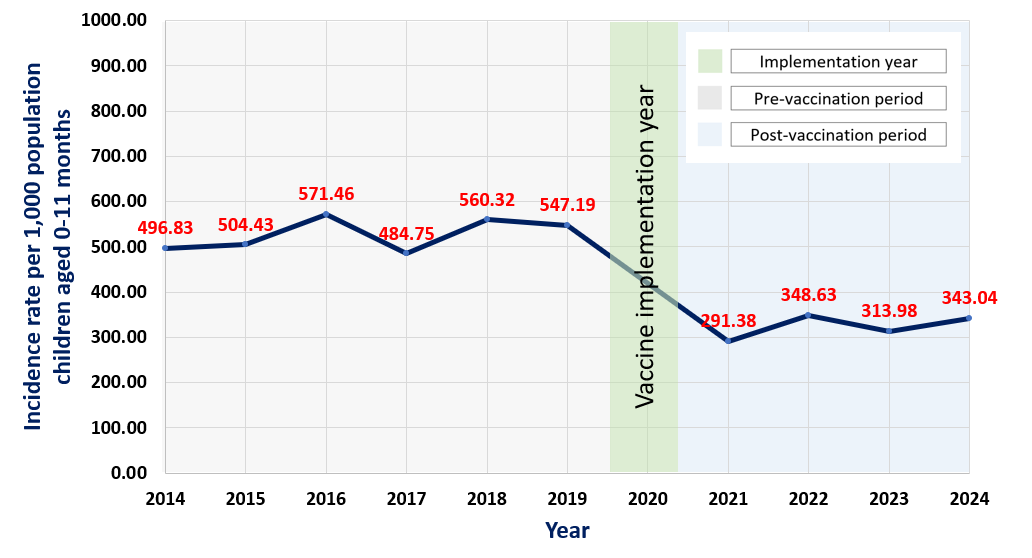
**

**Supplementary Figure S8.** Incidence of hospitalisation due to all-cause acute diarrhoea in children aged 0-11 months.

**
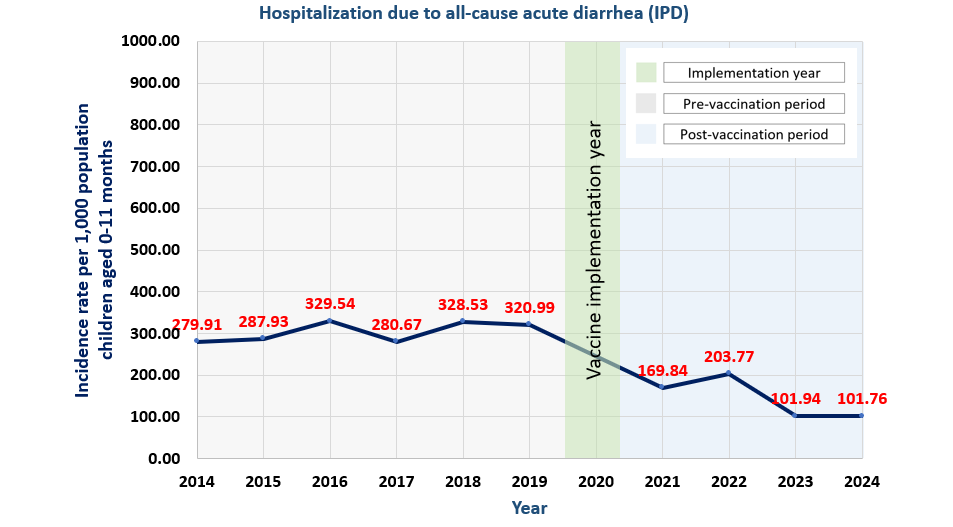
**

**Supplementary Figure S9.** Incidence of all-cause acute diarrhoea in children by age group covered by vaccination and not covered by vaccination program.

**
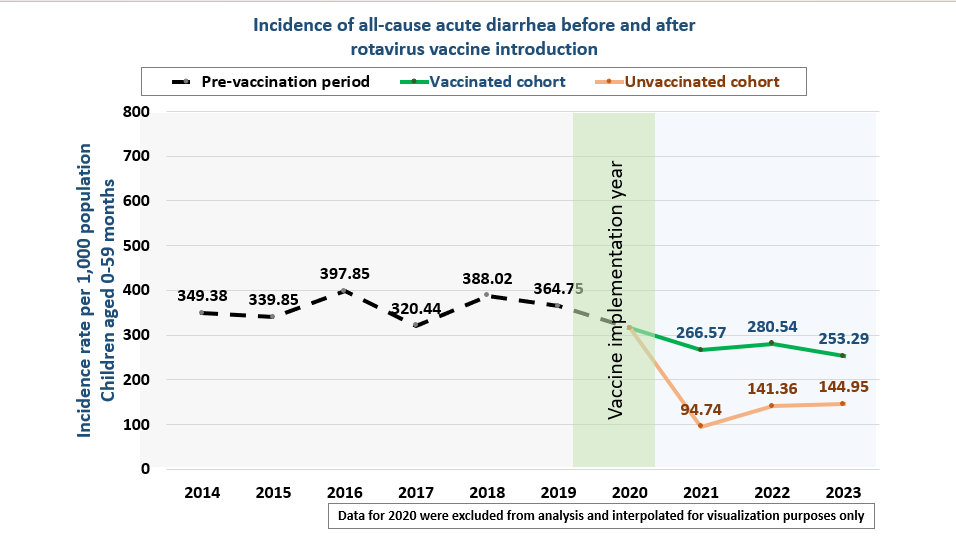
**

**Supplementary Figure S10.** Incidence of hospitalisation due to all-cause acute diarrhoea by age group covered by vaccination and not cover by vaccination program.

**
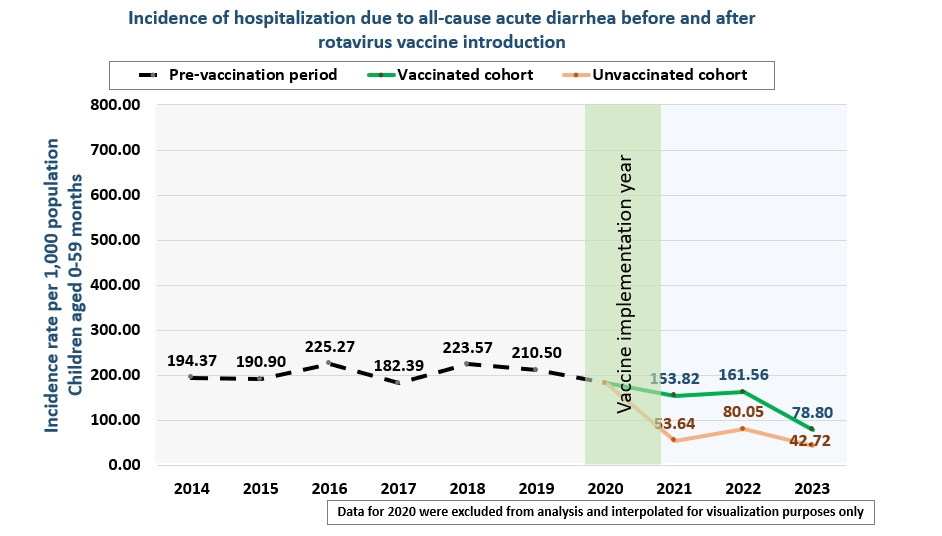
**

**Supplementary Figure S11.** Incidence of intussusception in children aged 0-11 months.

**
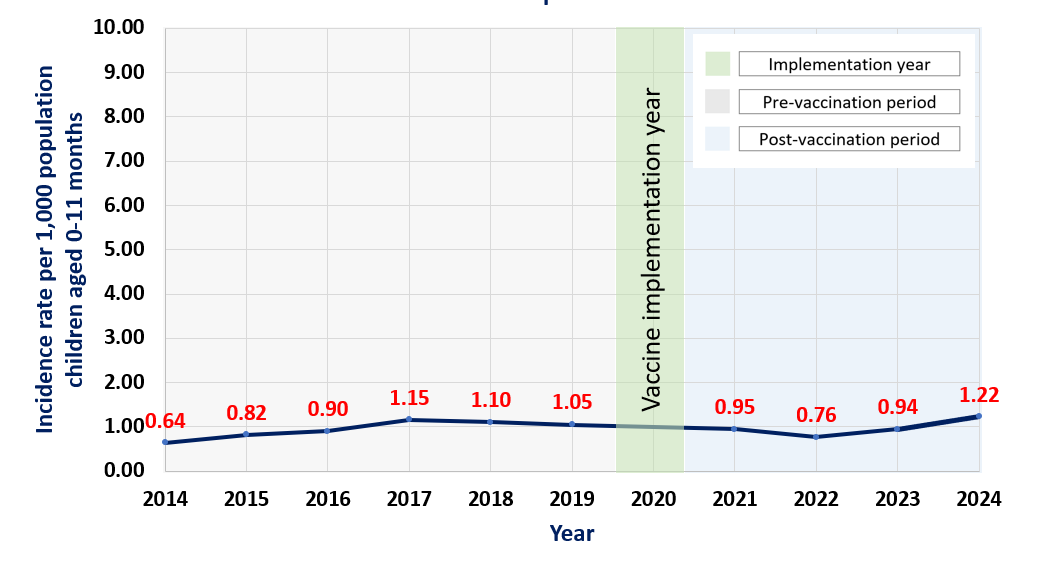
**
