# Supplementary material for: Incidence of acute diarrhoea among children (0–59 months old) in Thailand after introduction of rotavirus vaccine: a retrospective national database analysis (2014–2024)
Source: Lancet Reg Health Southeast Asia. 2026 Jun 3;50:100793. doi: 10.1016/j.lansea.2026.100793 (PMC13254665; doi:10.1016/j.lansea.2026.100793)
Supplement: Translated Abstract [file mmc2.docx]

***Disclaimer***

***This translation in Thai was submitted by the authors, and we reproduce it as supplied. It has not been peer reviewed. Our editorial processes have only been applied to the original abstract in English, which should serve as reference for this manuscript.***

**ผลของการใช้วัคซีนโรต้าไวรัสในแผนการสร้างเสริมภูมิคุ้มกันโรคแห่งชาติต่อภาวะท้องเสียเฉียบพลันในเด็กไทย: การวิเคราะห์ฐานข้อมูลระดับประเทศแบบย้อนหลัง (พ.ศ. 2557–2567)**

**บทนำ:** วัคซีนโรต้าไวรัสสามารถลดภาระโรคท้องเสียในเด็กได้ อย่างไรก็ตามข้อมูลการติดตามผลระดับประเทศจากประเทศที่มีทรัพยากรจำกัดยังมีไม่มาก การศึกษานี้มีวัตถุประสงค์เพื่อประเมินผลของการบรรจุวัคซีนโรต้าไวรัสในแผนการสร้างเสริมภูมิคุ้มกันโรคแห่งชาติของประเทศไทย ในเด็กอายุ 0–59 เดือน (วัคซีนชนิด 1 สายพันธุ์: กุมภาพันธ์ 2563–สิงหาคม 2564; ชนิด 5 สายพันธุ์: สิงหาคม 2564–กุมภาพันธ์ 2566; ชนิด 1 สายพันธุ์: ตั้งแต่กุมภาพันธ์ 2566 เป็นต้นไป)

**วิธีการศึกษา:** วิเคราะห์ข้อมูลระดับประเทศ (ICD-10-TM: A00–A09, K56·1) จากระบบคลังข้อมูลด้านการแพทย์และสุขภาพ (Health Data Center, HDC) ครอบคลุมร้อยละ 90 ของสถานพยาบาลรัฐในสังกัดกระทรวงสาธารณสุข รวมข้อมูลผู้ป่วยนอก ผู้ป่วยใน และการเสียชีวิต คำนวณอัตราป่วยและอัตราการนอนโรงพยาบาลจากโรคท้องเสีย, เปรียบเทียบอุบัติการณ์โรคท้องเสียในเด็กอายุ 0–59 เดือนและอัตราการเกิดภาวะลำไส้กลืนกันในเด็กอายุ 0–11 เดือน ในช่วงก่อนการให้วัคซีน (พ.ศ. 2557–2562) และหลังการให้วัคซีน (พ.ศ. 2564–2567) โดยไม่นับรวมปี พ.ศ. 2563 ซึ่งเป็นปีที่เริ่มมีการให้วัคซีนรวมถึงวิเคราะห์จำแนกตามกลุ่มอายุเพื่อใช้เป็นตัวแทนของระดับการได้รับวัคซีนที่แตกต่างกันในแต่ละกลุ่ม

**ผลการศึกษา:** หลังการใช้วัคซีนโรต้าไวรัส อุบัติการณ์ของท้องเสียเฉียบพลันจากทุกสาเหตุและจากโรต้าไวรัสลดลงร้อยละ 50·0 (IRR 0·50 [95%CI 0·39–0·63]) และร้อยละ 52·0 (IRR 0·48 [0·39–0·59]) ตามลำดับ อัตราการนอนโรงพยาบาลจากท้องเสียเฉียบพลันจากทุกสาเหตุและจากโรต้าไวรัสลดลงร้อยละ 58·2 (IRR 0·42 [0·30–0·58]) และร้อยละ 49·0 (IRR 0·51 [0·30–0·88]) ภายใน 1–4 ปีหลังเริ่มโครงการ อัตราการเสียชีวิตจากท้องเสียเฉียบพลันจากทุกสาเหตุไม่แตกต่างอย่างมีนัยสำคัญระหว่างช่วงก่อนและหลังการให้วัคซีน (IRR 0·94 [0·50–1·77]) ความรุนแรงของฤดูกาลระบาดช่วงเดือนธันวาคมถึงกุมภาพันธ์ลดลง และไม่พบการเพิ่มขึ้นของอัตราการเกิดภาวะลำไส้กลืนกันในช่วงหลังการให้วัคซีน (IRR 1·03 [0·82–1·29])

**สรุปผล:** การบรรจุวัคซีนโรต้าไวรัสในแผนการสร้างเสริมภูมิคุ้มกันโรคแห่งชาติในประเทศไทย สามารถลดภาระโรคท้องเสียในเด็กได้อย่างมีนัยสำคัญ การคงระดับความครอบคลุมของวัคซีนในระดับสูงยังคงเป็นสิ่งสำคัญเพื่อให้เกิดประโยชน์ด้านสาธารณสุขสูงสุด

**แหล่งทุน:** ไม่มี
